# Characterization of air pollution and associated health risks in Gansu Province, China from 2015 to 2022

**DOI:** 10.1038/s41598-024-65584-2

**Published:** 2024-06-26

**Authors:** Bowen Cheng, Yuxia Ma, Pengpeng Qin, Wanci Wang, Yuhan Zhao, Zongrui Liu, Yifan Zhang, Linbo Wei

**Affiliations:** College of Atmospheric Sciences, Key Laboratory of Semi-Arid Climate Change, Ministry of Education, Lanzhou University, Lanzhou, 730000 China

**Keywords:** Air quality index, Air pollution, Health effects, Environmental impact, Risk factors

## Abstract

Air pollution poses a major threat to both the environment and public health. The air quality index (AQI), aggregate AQI, new health risk–based air quality index (NHAQI), and NHAQI-WHO were employed to quantitatively evaluate the characterization of air pollution and the associated health risk in Gansu Province before (P-I) and after (P-II) COVID-19 pandemic. The results indicated that AQI system undervalued the comprehensive health risk impact of the six criteria pollutants compared with the other three indices. The stringent lockdown measures contributed to a considerable reduction in SO_2_, CO, PM_2.5_, NO_2_ and PM_10_; these concentrations were 43.4%, 34.6%, 21.4%, 17.4%, and 14.2% lower in P-II than P-I, respectively. But the concentration of O_3_ had no obvious improvement. The higher sandstorm frequency in P-II led to no significant decrease in the ER_total_ and even resulted in an increase in the average ER_total_ in cities located in northwestern Gansu from 0.78% in P-I to 1.0% in P-II. The cumulative distribution of NHAQI-based population-weighted exposure revealed that 24% of the total population was still exposed to light pollution in spring during P-II, while the air quality in other three seasons had significant improvements and all people were under healthy air quality level.

## Introduction

Air pollution has emerged as a critical global environmental concern and has attracted considerable research attention^1,2^. Approximately 90% of the population over the world live in areas where the ambient air quality fails to meet the guidelines set by the World Health Organization (WHO), with the highest health burden on countries with low and middle incomes^3^. Air pollution is a prominent environmental concern in China, with more than 2 million deaths attributable to it annually^4^. Epidemiological studies have demonstrated the detrimental impacts of both short-term and long-term exposure to ambient air pollution on physical and mental health; these impacts include increased disease burden, higher mortality risks, and reduced life expectancy^5–7^.

Particulate matters (PMs) of PM_10_ and PM_2.5_ are the major health risk factors^8^, and exposure to PM_10_ and PM_2.5_ can lead to respiratory infections, emphysema, reduced lung function, and even chronic pulmonary heart disease^9,10^. Gaseous pollutants (O_3_, NO_2_, SO_2_, and CO) could irritate the respiratory tract, trigger inflammatory reactions, and potentially damage the kidneys, heart, and central nervous system^7,11^. Each pollutant has distinct health effects, making it crucial to assess the cumulative distribution of population exposure to air pollution and quantify the health risks in specific regions. The air quality index (AQI) is widely used to assess air quality. The AQI is based on the individual pollutant with the highest concentration among the six pollutants mentioned above, and it may underestimate the actual air pollution levels, particularly during heavy pollution events^12,13^. The aggregate air quality index (AAQI)^14^ and the health risk-based air quality index (HAQI)^13^ were developed to consider the comprehensive impacts of all the pollutants. In this study, health risk was defined as the risk of mortality. Compared with the AQI, these new indices provide a more accurate assessment of the combined health risk effects of the six criteria pollutants^15^. Thus, employing these indices to assess the quality characterization of pollution and the related health effect on the basis of the spatial and temporal variability of ambient air pollutants is essential.

Since the global spread of COVID-19, numerous studies illuminated a substantial link between air pollution and COVID-19^16–18^. In response to the COVID-19 pandemic, governments worldwide imposed strict restrictions and lockdowns, which resulted in the cessation of numerous economic activities and the reduction of anthropogenic emissions of air pollutants^19^. A study conducted in the United Kingdom^20^ reported a remarkable 69% reduction in traffic volume during the COVID-19 lockdown period, which led to a decrease of 38.3% and 16.5% in NO_2_ and PM_2.5_, respectively. A study of 325 Chinese cities^21^ reported that the averted premature deaths estimated to be in the range 26,385–38,977 due to the COVID-19 pandemic. The rapid decline in anthropogenic emissions and economic activities during the pandemic has offered a unique chance to investigate the impact of natural interventions on air pollutants and offer guidance for future clean air initiatives.

Air quality was significantly different before and after COVOD-19. We divided the study period into two parts: Period I (P-I), covering the years 2015–2019 before the COVID-19 pandemic, and Period II (P-II), encompassing the years 2020–2022 after the COVID-19 outbreak. Using four indices, we quantitatively assessed the characterization of different air pollution and the health effect in Gansu Province before and after COVID-19. The results of this study have implications for future clean air initiatives and environmental management policies.

## Methods

### Study region

**Figure 1:**
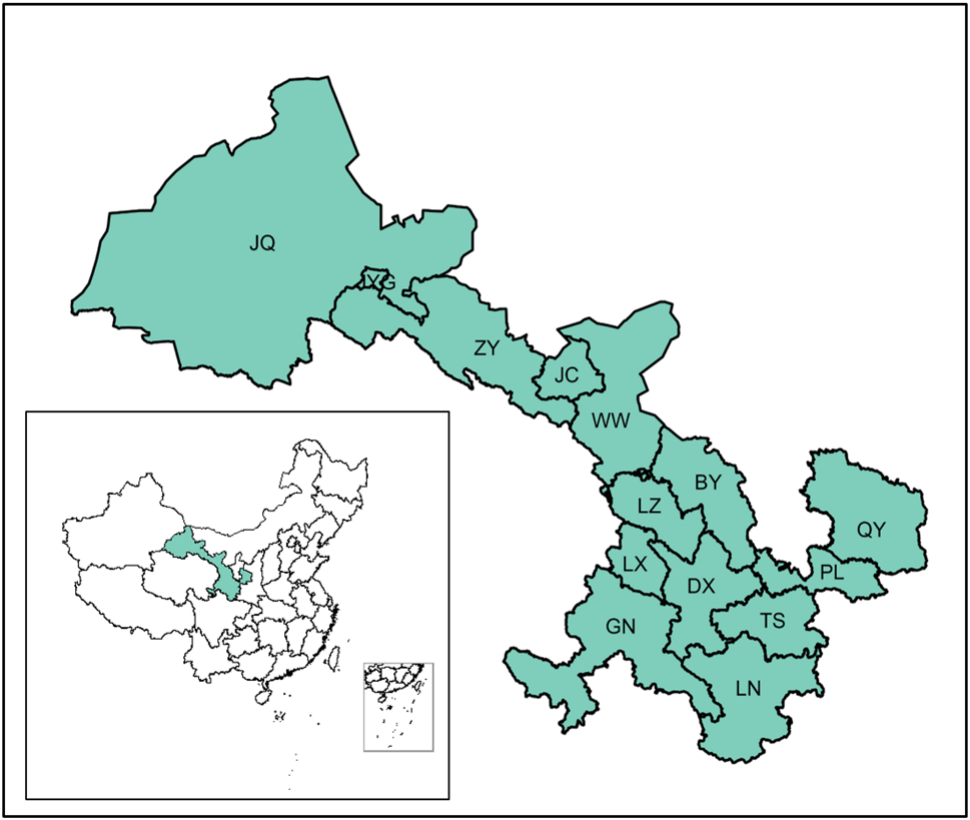
Map of Gansu Province (R version 4.0.1 https://www.r-project.org/).

Gansu Province is located at the confluence of the Loess Plateau, the Inner Mongolian Plateau and the Tibetan Plateau, which is a unique and diverse region. Additionally, Gansu Province is located on the northeastern edge of the Tibetan Plateau, and its topography is mainly influenced by the substantial uplift of the Tibetan Plateau. Consequently, the terrain descends in a stepped pattern from the south to the north and from the west to the east^22^. The main industries in Gansu Province are the energy and materials industries, including nonferrous metals, petrochemicals, electric power, metallurgy, machinery manufacturing, and building materials^23^. Figure 1 depicted 14 cities in Gansu Province (Baiyin [BY], Dingxi [DX], Gannan [GN], Jiayuguan [JYG], Jinchang [JC], Jiuquan [JQ], Lanzhou [LZ], Linxia [LX], Longnan [LN], Pingliang [PL], Qingyang [QY], Tianshui [TS], Wuwei [WW], and Zhangye [ZY]).

### Data sources

Data on the daily concentration of PM_2.5_, PM_10_, SO_2_, NO_2_, and CO and on the 8-h average O_3_ for the 14 cities in Gansu Province were provided by the National Environmental Monitoring stations for the period January 1, 2015, to December 31, 2022. The pollutant data for each city were the average concentration from stations in the city. The data of population and mortality in Gansu Province for the period 2015–2022 used in the exposure assessment were supplied by the Statistical Yearbook of Gansu Province (http://tjj.gansu.gov.cn/tjj/c109464/info_disp.shtml).

### Air quality index

The Ministry of Environmental Protection (MEP) in China employs the AQI as a metric to assess ambient air quality on the basis of the Chinese Ambient Air Quality Standards (CAAQS). The respective Air Quality Index (AQI_*i*_) for each individual pollutant was obtained by Eq. (1), and the maximum value among the sub-AQI_*i*_ was selected as overall AQI, as indicated in Eq. (2):

$${AQI}_{i}=\frac{({AQI}_{i,j}-{AQI}_{i,j-1})}{({C}_{i,j}-{C}_{i,j-1})}\times \left({C}_{i,m}-{C}_{i,j-1}\right)+{AQI}_{i,j-1}, j>1$$

1 $${AQI}_{i}={AQI}_{i,1}\times \frac{{C}_{i,m}}{{C}_{i,1}}, j=1$$

2 $$AQI=\text{max}\left\{{AQI}_{1},{AQI}_{2},\dots ,{AQI}_{n}\right\}, n=\text{1,2},\dots ,6$$

where *i* refers to pollution *i*; *j* represents health category index; AQI_*i*_ indicates the air quality index of pollution *i*; AQI_*i,j*_ and AQI_*i,j*−1_ are the AQI values of pollution *i* corresponding to the *j*th and (*j* − 1)th health type, respectively; C_*i,m*_ refers to the observation concentration of *i*; and C_*i,j*_ and C_*i,j*−1_ are the upper limit pollution *i*. Table S1 shows reference AQI values and concentrations of each pollution in different health type according to the Chinese MEP^24^.

### Aggregate air quality index

In order to consider the comprehensive air quality effects of exposure to air pollution, the AAQI was proposed^14^:

3 $$AAQI={\left({\sum }_{i=1}^{n}{({AQI}_{i})}^{\rho }\right)}^{\frac{1}{\rho }}$$

where AQI_*i*_ refers to the air quality index of pollutant *i*, and *ρ* refers to a constant of experience. The choices for ρ was ambiguous, whereas studies indicated that the optimal range is chosen between 2 and 3^25^. The *ρ* value of 2.0 was selected for the current study. For a more comprehensive comparison with the AQI, the AAQI adopts the same health type as the AQI and has a scale range of 0–500, with an upper limit of 500 set for the AAQI when values exceed 500.

### Health risk-based indices

The health risk-based index (HAQI) was proposed to greater demonstrate the connection related to air pollutants and their health risk^8,13^. The HAQI incorporates the concept of total excess risk (ER) associated with exposure to multiple air pollutants^25^. First, the relative risk (RR_*i*_) for pollution *i* is obtained by Eq. (4):

4 $${RR}_{i}=\mathit{exp}\left[{\beta }_{i}({C}_{i,m}-{C}_{i,0})\right],{C}_{i,m}>{C}_{i,0}$$

where β_*i*_ refers to the exposure–response relationship coefficient, denoting the excess health risk associated with each unit increase of pollution *i*. The *β* values were shown in Table S2 according to a meta-analysis conducted on China^26^. *C*_*i,m*_ represents the observation concentration of *i*, and *C*_*i*,0_ donates the risk limit of pollutant *i*. In this study, *C*_*i*,0_ denotes the upper limit value of the 24-h Grade II CAAQS, and it was assessed to indicate no excess health effect when the concentration of pollutant *i* was less than *C*_*i*,0_. Furthermore, the new WHO Air Quality Guidelines (WHO AQG2021)^27^ were employed as thresholds to calculate the NHAQI-WHO and ER (Table S3).

The ER_*i*_ of each pollutant is obtained by Eq. (5):

5 $${ER}_{i}={RR}_{i}-1$$

The total ER of concurrent exposure to all the pollutants is the sum of the ER_*i*_ values of each pollutant:

6 $${ER}_{total}={\sum }_{i=1}^{n}{ER}_{i}={\sum }_{i=1}^{n}({RR}_{i}-1)$$

Hu et al.^13^ proposed the equivalent concentration for calculating the HAQI based on the same scale of the AQI and AAQI (0–500), where C^*^_i,m_ denotes the equivalent concentration of pollutant *i* when the value of ER_*i*_ is equal to ER_total_. Ma et al.^28^ indicated that the use of C^*^_i,m_ in the calculation may lead to a higher HAQI value and improved the HAQI computation by using the partition function. Thus, C^*^_i,m_ is obtained by Eq. (8) when there was an excess health risk; otherwise, it was still the observation concentration of pollutant *i*. The equivalent relative risk (RR_i_^*^) is calculated according to Eq. (7):

7 $${RR}_{i}^{*}={ER}_{total}+1=\text{exp}[{\beta }_{i}(({C}_{i,m}-{C}_{i,0}))]$$

C^*^_i,m_ is calculated according to Eq. (8) and (9):

8 $${C}_{i,m}^{*}=\text{ln}({RR}_{i}^{*})/{\beta }_{i}+{C}_{i,0}, {C}_{i,m}>{C}_{i,0}$$

9 $${C}_{i,m}^{*}={C}_{i,m}, {C}_{i,m}<{C}_{i,0}$$

The new health risk–based index (NHAQI) is constructed by substituting the equivalent concentration for the observation concentration, as shown below:

$${NHAQI}_{i}=\frac{({AQI}_{i,j}-{AQI}_{i,j-1})}{({C}_{i,j}-{C}_{i,j-1})}\times \left({C}_{i,m}^{*}-{C}_{i,j-1}\right)+{AQI}_{i,j-1}, j>1$$

10 $${NHAQI}_{i}={AQI}_{i,1}\times \frac{{C}_{i,m}^{*}}{{C}_{i,1}}, j=1$$

11 $$NHAQI=\text{max}\left\{{NHAQI}_{1},NH{AQI}_{2},\dots ,{NHAQI}_{n}\right\}, n=\text{1,2},\dots ,6$$

### Mortality burden estimation

The mortality burden during the two periods in Gansu Province is evaluated as follows:

12 $$\Delta \text{Mortality}=ER\times POP\times {Y}_{b}$$

where ∆Mortality refers to the additional deaths attributed to pollution, *ER* is the percentage change of the mortality rate exposure to air pollution, *POP* indicates the exposure population, and *Y*_*b*_ represents the baseline total mortality. A significant change in the total mortality rate from 2020 to 2023 was noted due to the influence of the COVID-19. Therefore, the average total mortality risk from 2015 to 2019 was employed to substitute for P-II.

## Results

Table 1 indicates the air pollution concentration in Gansu in P-I and P-II. The annual mean data indicate that air quality was significantly better in P-II than in P-I. There was a considerable reduction in SO_2_, CO, PM_2.5_, NO_2_ and PM_10_ and these concentrations were 43.4%, 34.6%, 21.4%, 17.4%, and 14.2% lower in P-II than P-I, respectively. Notably, the concentration of O_3_ was not obviously improved in P-II, with the O_3_ concentrations found to be higher in half of the cities. Natural pollutant sources such as dust storms have a considerable impact on the cities in northwest Gansu (JC, JQ, JYG, WW, and ZY), and PM_10_ and PM_2.5_ in these cities only decreased by 3.9% and 5.9% in P-II compared with P-I, with even increases in PM concentrations in some cities. Compared with cities in northwestern Gansu, cities in southeastern Gansu experienced greater improvements in air quality. The concentrations of SO_2_ and CO were mostly decreased that met the Grade I CAAQS standards.

**Table 1:** Annual average air pollution concentration of Gansu in P-I and P-II.

City	PM_2.5_		PM_10_		SO_2_		NO_2_			O_3_			CO		
	(μg/m^3^)		(μg/m^3^)		(μg/m^3^)		(μg/m^3^)			(μg/m^3^)			(mg/m^3^)		
	I	II	I	II	I	II	I		II	I		II	I		II
BY	37.8	30.4	101.4	87.4	44.0	30.7	26.7		22.8	87.5		92.5	0.9		0.7
DX	36.8	26.8	81.6	66.5	20.2	10.1	27.1		23.2	95.0		95.0	0.7		0.5
GN	32.8	18.4	67.2	40.7	16.4	10.0	21.3		18.6	101.1		94.8	0.9		0.5
JC	29.1	24.3	97.1	90.3	28.0	17.1	16.4		15.6	102.5		97.9	0.8		0.6
JQ	37.2	31.2	119.2	105.3	13.5	7.5	27.3		21.9	100.5		100.1	0.6		0.5
JYG	28.5	29.6	95.5	93.0	16.2	14.5	25.8		20.2	106.2		98.6	0.6		0.5
LN	28.3	18.1	54.8	43.3	18.7	13.2	23.6		19.2	83.0		84.5	0.8		0.5
LX	42.6	29.5	84.8	70.2	24.5	8.6	32.2		24.7	96.4		98.3	1.2		0.7
LZ	48.5	35.9	118.9	91.8	20.1	15.2	54.0		41.0	96.1		99.3	1.2		0.9
PL	36.8	23.4	82.2	64.0	14.9	7.2	38.4		32.5	92.3		93.9	0.8		0.5
QY	35.4	28.8	76.9	64.5	24.8	9.2	20.1		15.0	95.4		97.6	0.9		0.7
TS	39.0	27.6	79.1	58.1	22.2	10.1	34.6		26.2	89.9		88.0	0.8		0.6
WW	40.2	39.1	104.0	109.8	16.9	7.2	27.0		23.0	98.1		100.6	1.1		0.5
ZY	35.4	36.1	86.6	86.1	21.0	9.7	20.8		22.2	105.9		99.3	0.6		0.4
Average	36.3	28.5	89.2	76.5	21.5	12.2	28.2		23.3	96.4		95.7	0.9		0.6
CAAQS Grade I/II	35/75		50/150		50/150		40/80			100/160			2/4		

**Figure 2:**
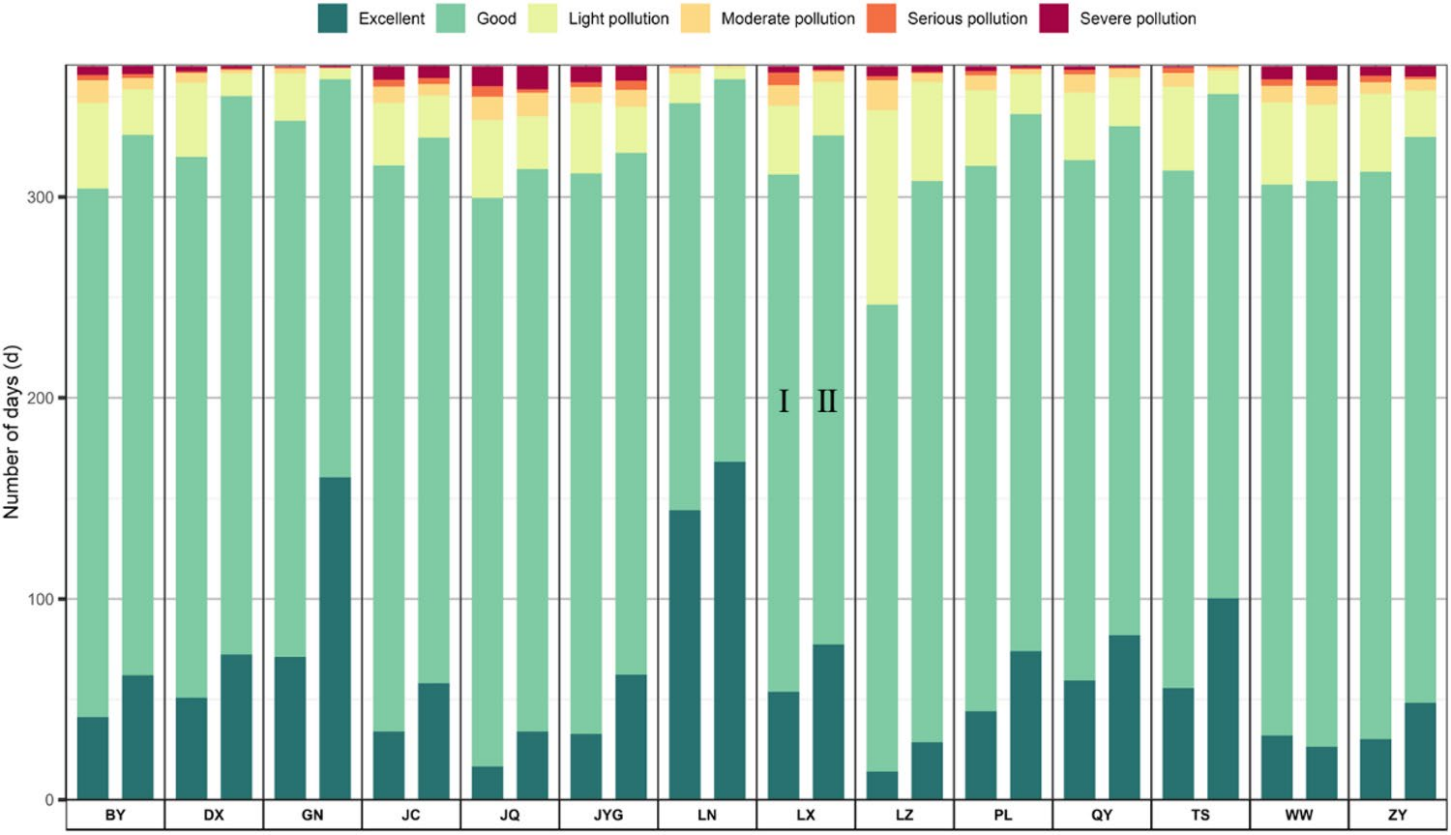
Cumulative days for each of the six AQI type in the 14 cities in the two Periods.

Figure 2 depicts cumulative days for each of the six AQI type in the 14 cities during the two Periods. Air quality in the 14 cities improved significantly, with excellent or good days increasing from 311.4 in P-I to 333.5 in P-II. The most significant improvements in air quality were observed in LZ, TS, DX, BY, and PL, where the excellent or good days increased by > 25. In particular, the most significant improvement in air quality was that for the provincial capital LZ, with days of excellent or good air quality increasing from 246.4 in P-I to 308.0 in P-II. Overall, the days of moderate, serious, or severe pollution levels was reduced from 14.7 days to 10.5 days. But, no obvious improvement was observed in the days with moderate, severe, or serious pollution in the cities of northwest Gansu, with even increases in the number of these days discovered for WW and JYG.

**Figure 3:**
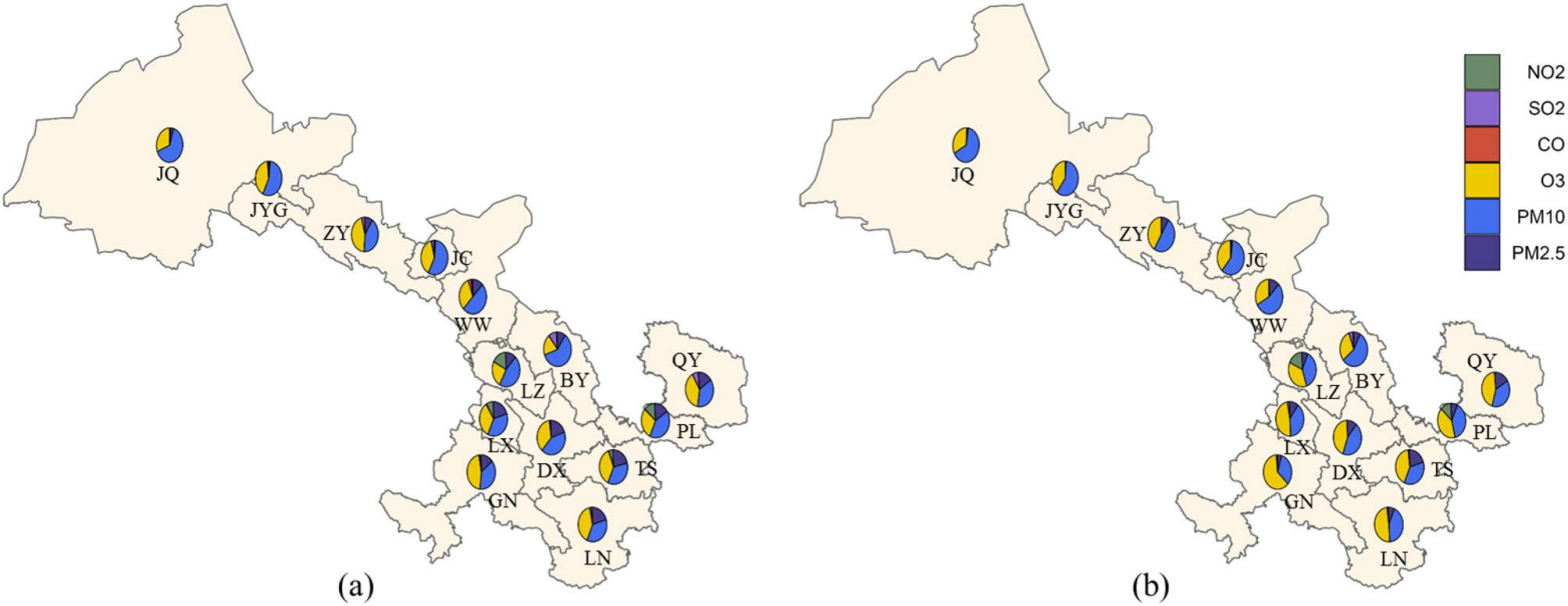
The number fractions of predominant pollution in the 14 cities (**a** for P-I; **b** for P-II) (R version 4.0.1 https://www.r-project.org/).

Figure 3 indicates the number fractions of predominant pollution in the 14 cities of Gansu Province during the two Periods. The predominant pollution was those with the highest AQI values when the AQI exceeded 50, as determined by Eq. (1). In both P-I and P-II, PM_10_, O_3_, and PM_2.5_ were the most frequent predominant pollution and accounted for more than 90% of the total pollutants. The proportion of PM_10_ exhibited a slight increase from 45.8% in P-I to 46.8% in P-II, whereas the proportion of O_3_ increased significantly from 35.2% in P-I to 41.4% P-II. However, a significant decrease in the percentage of PM_2.5_ was found, from 13.1% in P-I to 8.8% in P-II. LZ, LX, and PL had relatively high levels of NO_2_ pollution, indicating the specific characteristics of air pollution in these cities. In general, the proportions of SO_2_, NO_2_, and CO were in a low level and further reduced in during P-II. In particular, the proportions of CO and SO_2_ were < 1% in P-II. Additionally, significant seasonal variation was observed in the proportion of predominant pollutants (Fig. S1). During spring and autumn, PM_10_ and O_3_ were the predominant pollution for all cities, contributing more than 50% and 20% of the total pollutants, respectively. Moreover, northwestern cities have a higher proportion of PM_10_ than southeastern cities. During summer, O_3_ accounted for more than 75% of the total pollutants, making it the frequently dominant pollutant. By contrast, during winter, PM_10_ and PM_2.5_ were predominant pollution for the all province, contributing more than 25% and 50% of the total pollutants, respectively.

**Figure 4:**
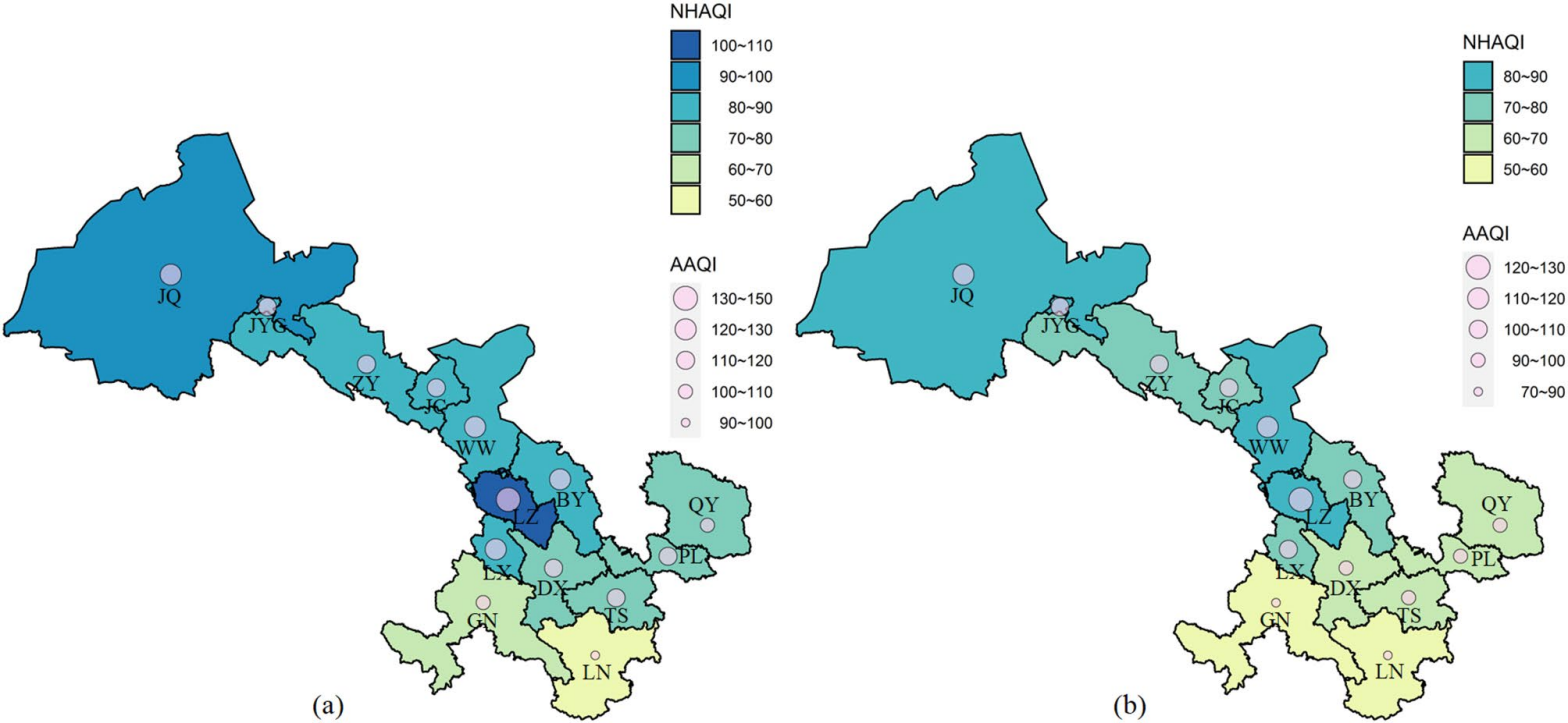
Regional variations of mean AAQI and NHAQI in Gansu (**a** for P-I; **b** for P-II) (R version 4.0.1 https://www.r-project.org/).

Figure 4 illustrates the regional variations of mean AAQI and NHAQI in Gansu. The air quality in southeastern Gansu was greater than that in northwestern Gansu. Specifically, during P-I, LZ exhibited the highest level of pollution, categorized as light pollution on the basis of the NHAQI, while all other cities had healthy air quality levels. During P-II, the most significant improvement in air quality was that for LZ, and on the basis of the NHAQI, all cities were classified under the healthy air quality level. Compared with the cities in the southeast of Gansu, most cities in the northwest (i.e., JQ, JYG, ZY, JC, and WW) had smaller improvements in air quality, with all improvements being < 10%. By contrast, the air quality of all cities in southeastern Gansu improved by > 10% except for LN, which according to the AAQI already had the best air quality in P-I. During P-II, most cities in southeastern Gansu (i.e., DX, GN, LN, PL, QY, and TS) were classified by the AAQI as being under the healthy air quality level, whereas the cities in the northwest were all classified as having a light pollution level.

**Figure 5:**
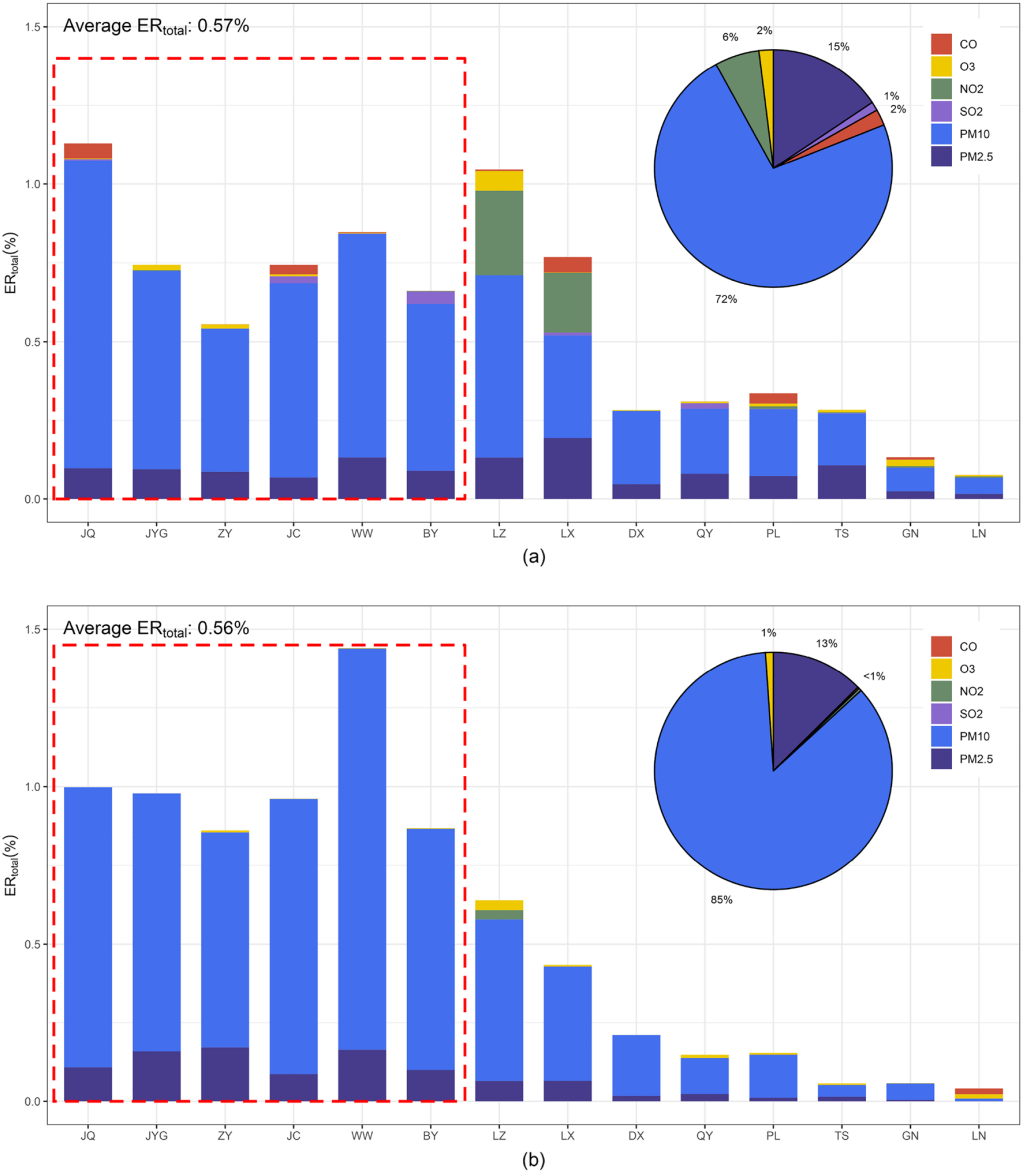
Average ER_total_ in Gansu (**a** for P-I; **b** for P-II; the red dotted box is the northwestern cities of Gansu).

Figure 5 depicts the average total excess risk (ER_total_) in Gansu. During P-I, ER_total_ of Gansu was 0.57%, with considerable differences in ER_total_ between cities. The ER_total_ values for JC, JQ, JYG, LX, LZ, and WW were high, all exceeding 0.7%. GN and LN had the best air quality, with ER_total_ < 0.15%. The main pollutants contributing to ER_total_ were PM_2.5_ and PM_10_, accounting for 72.4% and 12.6% of the ER_total_ from the six criteria pollutants, respectively. NO_2_ contributed only 6.0% to ER_total_, but LX and LZ were highly influenced by NO_2_. During P-II, the average ER_total_ value was 0.56%, indicating little change compared with P-I. However, significant differences were discovered between the northwestern and southeastern cities of Gansu Province. Due to the influence of dust storms, the average ER_total_ in the cities of northwestern Gansu (BY, JC, JQ, JYG, WW, and ZY) increased from 0.78% during P-I to 1.0% during P-II. By contrast, ER_total_ in the cities of southeastern Gansu Province (DX, GN, LN, LX, LZ, PL, QY, and TS) decreased from 0.40% during P-I to 0.22% during P-II.

**Figure 6:**
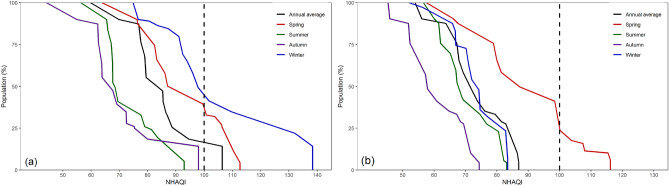
Cumulative distribution of population-weighted estimations based on average NHAQI in Gansu (**a** for P-I; **b** for P-II).

The population statistics of the cities were combined with the NHQI to determine cumulative distribution of population-weighted during P-I and P-II to more accurately quantify the proportion of population exposure to air pollutants (Fig. 6). On annual average, 17% of the public in Gansu were exposed to light pollution in P-I (100 < NHAQI < 150). The severity of air pollution varied across seasons, with the pollution most severe in spring and winter. In spring, 39% of the public in Gansu were affected by light pollution, whereas in winter, this proportion was 45%. By contrast, the air quality was better during summer and autumn, with all the public experiencing the health condition (NHAQI < 100). In P-II, air quality was significantly better than that in P-I, and for summer, autumn, and winter, the NHAQI indicated that air quality levels were healthy with no excess health risks. However, 24% of the total population still remained exposed to light pollution in the spring.

**Figure 7:**
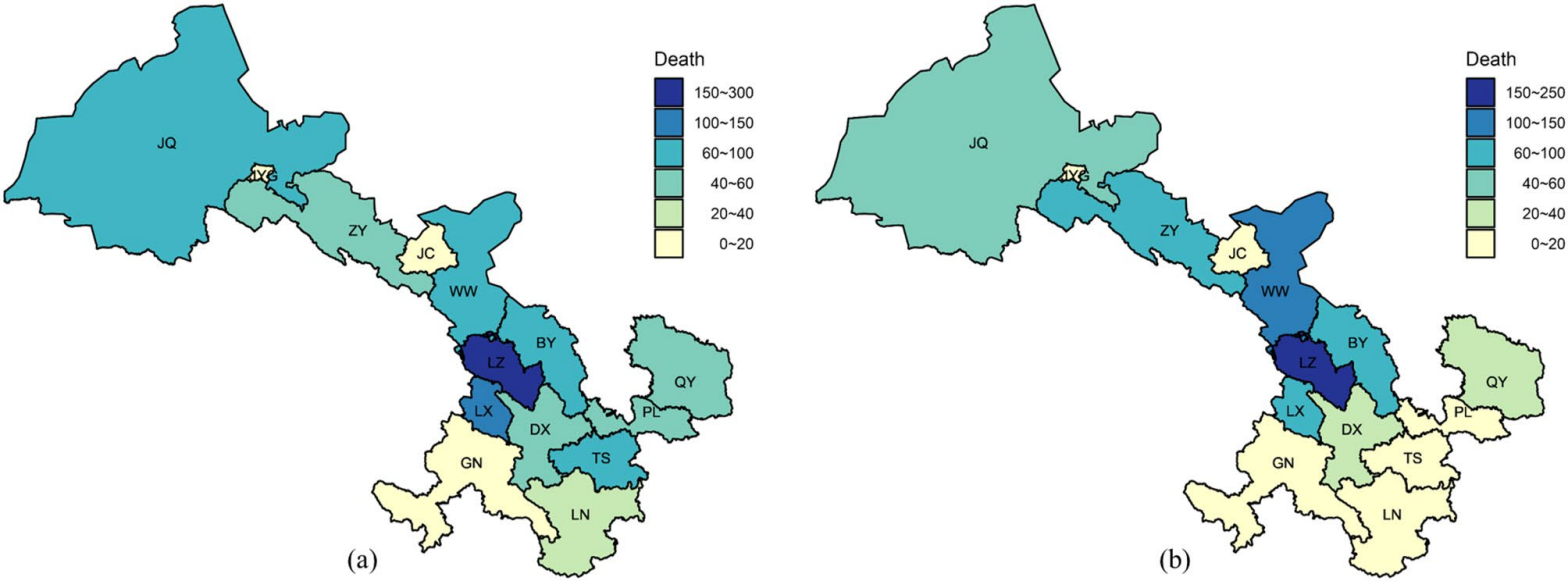
Regional variations of the annual average deaths due to air pollution in Gansu (**a** for P-I; **b** for P-II) (R version 4.0.1 https://www.r-project.org/).

Figure 7 presents the regional variations of the annual average deaths due to air pollution in Gansu. During P-I, a total of 938 annual average deaths were attributed to air pollution. LZ had the highest deaths due to air pollution, primarily caused by its severe air quality and high population density. Overall, in P-II, the annual average deaths due to air pollutants in whole province decreased to 721 (a 23.2% decrease), but significant differences in the changes were still observed between cities. Most of the cities in northwest Gansu (BY, JC, JQ, JYG, WW and ZY) had an increase in the number of deaths due to air pollution. The total number of deaths due to air pollution in the cities of southeastern Gansu (DX, GN, LN, LX, LZ, PL, QY, and TS) decreased from 650 during P-I to 374 during P-II.

Figures S2–S6 present the ER and NHAQI based on the WHO Air Quality Guidelines 2021, which are more stringent thresholds compared with the CAAQS-based values. According to the NHAQI-WHO, during P-I, LN had the best air quality with healthy air, whereas the other cities were under unhealthy environment with LZ having the most serious pollution and in the level of moderate pollution. During P-II, four cities in southeastern Gansu (DX, GN, LN, and QY) were under healthy air quality levels, but the other cities retained the light level of pollution. The ER and air pollution–related deaths based on the WHO AQG2021 thresholds were significantly higher than those based on the CAAQS-based values. The contributions of O_3_, NO_2_, and SO_2_ to the ER_total_-WHO increased, whereas the contribution of CO to the ER_total_-WHO still remained low. Regarding the cumulative distribution of the population-weighted exposure, according to the NHAQI-WHO, 97% of the public experienced unhealthy air pollution level during P-I. Additionally, 64% and 19% of the public were exposed to moderate and serious pollution levels, respectively. During P-II, 69% of the total population were still exposed to unhealthy air quality levels. Spring and winter were the seasons most severely affected by pollution, with the pollution being highest in winter.

## Discussion

China implemented stringent lockdown measures during the COVID-19 pandemic, and these measures resulted in significant improvements in air quality relating to PM_2.5_, PM_10_, SO_2_, NO_2_, and CO but not O_3_. During the pandemic, human activities such as industrial processes, transportation, and tourism decreased^29^. The public began to consciously maintain social distances, minimize social gatherings, avoid crowded places, and limit nonessential outings^30,31^. The reduction of human activity and industrial production would lead to a significant reduction in carbon emissions and energy consumption^32^. The total emissions from major polluting enterprises fell by 24.7%, with the mining, electric power, manufacturing, and natural gas industries being the most severely affected^33^. In particular, the transportation sector in China was directly affected by the epidemic, with a greater impact on the passenger sector than on the freight sector. Moreover, the outputs of the road transport, railroad, and aviation sectors decreased by 10.2%, 1.8%, and 1.5%, respectively^34,35^. The outbreak of COVID-19 provided a special time window to assess the impact of large-scale emission reduction on pollutant concentrations and regional air quality, and to explore the theoretical implications of reducing the health risks of air pollutants. This study can provide a scientific basis for effective air quality management and protection of public health in the future.

The concentration of O_3_ in was not considerably changed after the COVID-19 outbreak and even increased in seven cities in Gansu Province. The concentration of HCHO still remained stable in most urban areas of China, providing sufficient fuel for the production of tropospheric O_3_^31^. During the daytime, the increase in O_3_ caused by NO_x_ reduction offsets the decrease in O_3_ caused by a reduction in volatile organic compound (VOC) emission in the NO_x_ saturated region; during the nighttime, the titration of NO and the weakening of VOCs lead to an increase in O_3_ levels^36,37^. A weakened scattering effect due to the reduction of PMs may increase the photolysis rate and the reduced removal of HO_2_ caused by the lower fine particle loading may lead to an increase in O_3_ levels^38^. The significant increase in O_3_ concentrations during lockdown can be attributed to the enhancement of atmospheric oxidation capacity^39^ and the reduction of NO_x_^40^. Meteorological elements are also important factors affecting air pollution^41,42^. A study in nine cities worldwide^43^ indicated that low wind speeds, increase in maximum temperatures, and decrease in relative humidity would also lead to the increase in O_3_ during COVID-19. Regarding long-term trends, the annual average concentration of O_3_ in China significantly increased at a rate of 1.84 µg/m^3^ from 2013 to 2018 and peaked in 2018; O_3_ has become the second most abundant air pollutant after PM^44^. In Gansu Province, the number of summer days with O_3_ as the dominant pollutant increased from 77.8% in P-I to 89.2% in P-II. Therefore, future clean air plans should consider implementing synergistic measures for controlling both PM and O_3_. Decision makers should consider the complex relationships between different pollutants in establishing guidelines related to pollution control processes. And the control of O_3_ emissions required the coordination of NO_x_ and VOCs emissions.

The lower PM concentrations in P-II than in P-I in Gansu Province was largely attributable to the decrease in anthropogenic emissions. However, the extent of PMs decline varied significantly between the northwest and southeast regions of Gansu Province. Gansu Province is located downstream of the Taklamakan Desert, Kumtag Desert, and Tengger Desert^23,45^. The northwestern region of Gansu is located closer to the deserts, has low vegetation coverage, and therefore experiences dust storms significantly more frequently than the southeastern region of Gansu^46^. During spring and winter, the northwest region witnessed rapid increases in PM concentrations, especially PM_10_, as a result of frequent occurrence of sandstorms^47^. The changes in air pollutants at the geographical and temporal scales due to natural sources (mainly dust storms) were the major contributors of the regional variances in the concentration of PMs^8^. PM_2.5_ concentrations were high in winter due to the high levels of local emissions and poor dispersion, which were caused by the combustion of coal for heating, stabilization of weather symptoms, and maintenance of low boundary layer^48^.

Assessing the characterization of various air pollutants and their health effect is essential. PM is strongly associated with the increase in numerous diseases, especially severe viral respiratory diseases^49^. PM_2.5_ is easier to get into the lungs causing lung damage due to its smaller size^50^. A nationwide case-crossover study in China^51^ indicated that the incidence of out-of-hospital cardiac arrest increased by 2.37% and 2.12% for each interquartile range (IQR) increase in PM_2.5_ (27.91 μg/m^3^) and PM_2.5–10_ (22.03 μg/m^3^), respectively, with older people over 75 years more susceptible to the effects of PM_2.5–10_. The short-term effects of O_3_ can lead to respiratory inflammation, lung epithelial damage, and the increase of morbidity and mortality of lung cancer^52^. A study of multiple cities in China^53^ showed that on days with average temperatures higher than 75th, 85th, 90th and 95th percentiles, the risk of cardiovascular mortality increased by 0.74%, 0.76%, 0.80% and 1.11% for every 10 μg /m^3^ increase in O_3_-8 h, respectively. A study of short-term exposure to NO_2_^54^ demonstrated an IQR in NO_2_ concentrations (12.4 μg/m^3^) corresponded to a 13% and 17% increase in diabetes and dyslipidemia, respectively. A study in Guangzhou, China^55^ showed that ischemic and hemorrhagic stroke would increase by 1.27% and 1.55% for per 10 μg/m^3^ increase in the SO_2_. CO has a high affinity for haemoglobin and myoglobin, and exposure to CO would have negative effects on blood pressure, nervous system, and heart^56^. A study in 26 largest Chinese cities^57^ indicated that for every 1 mg/m^3^ increase in CO concentration, all-cause and cardiovascular admissions increased by 3.75% (95% CI 3.63–3.87%) and 4.39% (95% CI 4.07–4.70%), respectively.

The AAQI and NHAQI simultaneously evaluate the exposure–response relationships and the cumulative impact of all six criteria pollutants and thus offer a more comprehensive assessment^13,14^. A study conducted in the Changjiang River Basin, China^15^, indicated that, according to the HAQI and AAQI, 86% and 54% of days were under serious pollution level despite the pollution being classified as moderate on the AQI. The HAQI was widely used to assess variations in air pollution and health risk before and after COVID-19 pandemic. A study in China^58^ demonstrated that HAQI in 2020 was 14.4% lower than that in 2019 and identified PM_2.5_ as the most significant pollutant of the six criteria pollutants. Furthermore, on the basis of the ER and NHAQI-WHO, the thresholds for which are more stringent in the WHO AQG2021 values, air pollution presented a more serious risk to human health. Shen et al.^59^ demonstrated that the HAQI-WHO and HAQI-CAAQS for 31 Chinese provincial capitals decreased to 179 and 75, respectively (61% and 21% decreases) due to the COVID-19 pandemic but that the HAQI-WHO remained in the unhealthy category for public health. Lei et al.^60^ indicated that in China during 2019–2020, 40.0% of days with HAQI-WHO values > 100, whereas only 14.4% of days with AQI > 100, and highlighted the increasing importance of NO_2_ and O_3_ in air quality and health risk assessments based on the WHO AQG2021. The present study serves as a valuable scientific basis for future environmental management policies and the selection of air pollution thresholds.

The major contributors to ER were PM_10_ and PM_2.5_, particularly in P-II, for which their contributions were more than 95%. Although air quality in China has improved, PM pollution remains a major concern for both the environment and public health^61^. Liu et al.^62^ projected that PM_2.5_-related deaths would slightly decline to 1.23 million per year in 2023, but would not decline further by 2050 (1.21 million) due to the increasing age of China’s population. PM_2.5_ particles, being small in size, are more likely than other particles to penetrate deeply into the respiratory system, and due to their larger surface area and higher reactivity compared with PM_10_, they have high toxicity and relative risk^63,64^. The concentration of PM_10_ is significantly greater than that of PM_2.5_ in Gansu Province due to the region’s vulnerability to dust storms and the presence of mineral sources^65^. Northwest China experiences the greatest impact of PM pollution, with spring dust storms leading to the exceedance of PM_10_, making it the region most severely affected by PM pollution in the country^66^. A study conducted in northwest China also revealed that PM_10_ and PM_2.5_ were the main indicators of air pollution and contributed greatly to ER_total_ in Shaanxi, with PM_10_ dominating the health risk^28^. During P-II, the proportion of public exposed to air pollutants declined significantly than that in P-I for all seasons except spring, with the most notable improvement discovered in winter. However, an increase in dust storms led to a rise in the deaths in most cities in northwest Gansu. Dust storms in Gansu Province are more extensive and intense, as evident from the statistics of the Department of Ecology and Environment of Gansu Province^67,68^. The dust storm days in Gansu in 2020 and 2021 were 400 and 679, respectively, 45.5% and 146.9% more than in 2019. Severe dust storms from Mongolia sweeping through northern China in 2021 may indicate the beginning of an upward trend in dust storms, as the climate in inner East Asia has suddenly shifted to drier and hotter over the past two decades^69^. Due to the special natural environment, the northwest China region is more susceptible to natural sources of pollution than the eastern and central regions^70,71^. Dust storms consist of a complex mixture of elements, compounds, and pathogenic agents that can lead to various diseases. Ma et al.^66^ indicated that direct consumption of sandstorm particles is a major route through which humans are exposed to heavy metals, and the concentration of heavy metals in Lanzhou is five times higher than in Dunhuang during dust days. A study conducted in the Hexi Corridor, northwestern China^46^, demonstrated that the number of deaths from ischemic heart disease increased by 2.66% and 1.98% for males and females, respectively, for per IQR increase in the number of sandstorm days. Roy et al.^72^ indicated that dust storms can carry PM-bound bacteria at both the cancer-risk and non-cancer-risk levels, potentially constituting significant health risks to the respiratory system of humans. To mitigate the adverse effects of dust storms, measures that increase the surface vegetation cover, establish protective forest systems, and improve the ecological environment to facilitate windbreak and sand fixation should be implemented. The reduction of PM_10_ emissions is essential to reduce PM pollution and improve air quality in Gansu^73^.

This study has some limitations that should be considered when interpreting the results. First, a city’s pollution level was taken as the average of air pollution of the stations in the city, and this may have led to the spatial variability of air pollution within a city not being fully captured. Second, the HAQI used in this study assumed an independent contribution from each air pollutant to the health risks. However, in reality, interactions and synergistic effects between different pollutants may exist and influence the overall health impact. Future studies should explore these interactions to provide a more comprehensive and accurate assessment of health risks associated with air pollution. Third, the ER of PM is related to not only the mass concentration but also the chemical composition of that PM. Thus, the chemical composition should be fully considered in future research. Fourthly, in this study, we only considered the impact of COVID-19 lockdown on air quality, while other factors such as natural emissions, weather condition and governance policies should also fully consider.

## Conclusions

The study findings indicate that the strict lockdown measures implemented to control the spread of COVID-19 had a significant positive impact on air quality. PM is the main pollutant in Gansu Province, and PM contributed more than 85% of the ER_total_. Due to an increase in the frequency of dust storms, the reduction in PM concentrations in northwest Gansu was considerably smaller than that in southeast Gansu. The proportion of public exposed to air pollutants significantly declined in all seasons except spring during P-II, with the greatest improvement observed in winter. Compared with the other three indices, the AQI underestimates the comprehensive health risk impact. The results of this study provide a scientific basis for future air pollution control initiatives.

### Supplementary Information

Supplementary Information.

## Data Availability

The datasets used in the current study are available from the corresponding author on reasonable request.
