# Supplementary material for: Characterization of air pollution and associated health risks in Gansu Province, China from 2015 to 2022
Source: Sci Rep. 2024 Jun 26;14:14751. doi: 10.1038/s41598-024-65584-2 (PMC11208435; doi:10.1038/s41598-024-65584-2)
Supplement: Supplementary file 1 — Supplementary Information. [file 41598_2024_65584_MOESM1_ESM.docx]

Table S1. The health categories and the corresponding ranges of AQI values and pollutant concentrations.

| AQI | PM_2.5_ | PM_10_ | SO_2_ | NO_2_ | CO | O_3_ | Category | Health risks |
| --- | --- | --- | --- | --- | --- | --- | --- | --- |
|  | (μg/m^3^) | (μg/m^3^) | (μg/m^3^) | (μg/m^3^) | (mg/m^3^) | (μg/m^3^) |  |  |
| 0-50 | 35 | 50 | 50 | 40 | 2 | 100 | Excellent | Satisfactory, no risk |
| 51-100 | 75 | 150 | 150 | 80 | 4 | 160 | Good | Acceptable, may be a moderate risk for a very small number of people |
| 101-150 | 115 | 250 | 475 | 180 | 14 | 215 | Light pollution | Unhealthy for sensitive people (children, older adults, etc.) |
| 151-200 | 150 | 350 | 800 | 280 | 24 | 265 | Moderate pollution | Unhealthy (everyone begins to have adverse health effects) |
| 201-300 | 250 | 420 | 1600 | 565 | 36 | 800 | Serious pollution | Very unhealthy (everyone experience more serious health effects) |
| 301-400 | 350 | 500 | 2100 | 750 | 48 | 1000 | Very severe pollution | Hazardous (healthy people have significant symptoms) |
| 401-500 | 500 | 600 | 2620 | 940 | 60 | 1200 |  |  |

Table S2. The *β* values and 95% confidence intervals for each pollution (for each 1μg/m^3^ increase in PM_2.5_, PM_10_, SO_2_, NO_2_, and O_3_ and each 1mg/m^3^ increase in CO)

| Health endpoints | Pollution | β values |
| --- | --- | --- |
| Total mortality | PM_2.5_ | 0.038 (0.031, 0.045) |
|  | PM_10_ | 0.032 (0.028, 0.035) |
|  | SO_2_ | 0.081 (0.071, 0.091) |
|  | NO_2_ | 0.130 (0.119, 0.141) |
|  | O_3_ | 0.048 (0.038, 0.058) |
|  | CO | 3.7 (2.88, 4.51) |

Table S3. Threshold concentrations of pollutants based on WHO and CAAQS

|  | WHO AQG2021 (μg/m^3^) | CAAQS Grade II (μg/m^3^) |
| --- | --- | --- |
| PM_2.5_ | 15 | 75 |
| PM_10_ | 45 | 150 |
| SO_2_ | 40 | 150 |
| NO_2_ | 25 | 80 |
| O_3_ | 100 | 160 |
| CO | 4 (mg/m^3^) | 4 (mg/m^3^) |


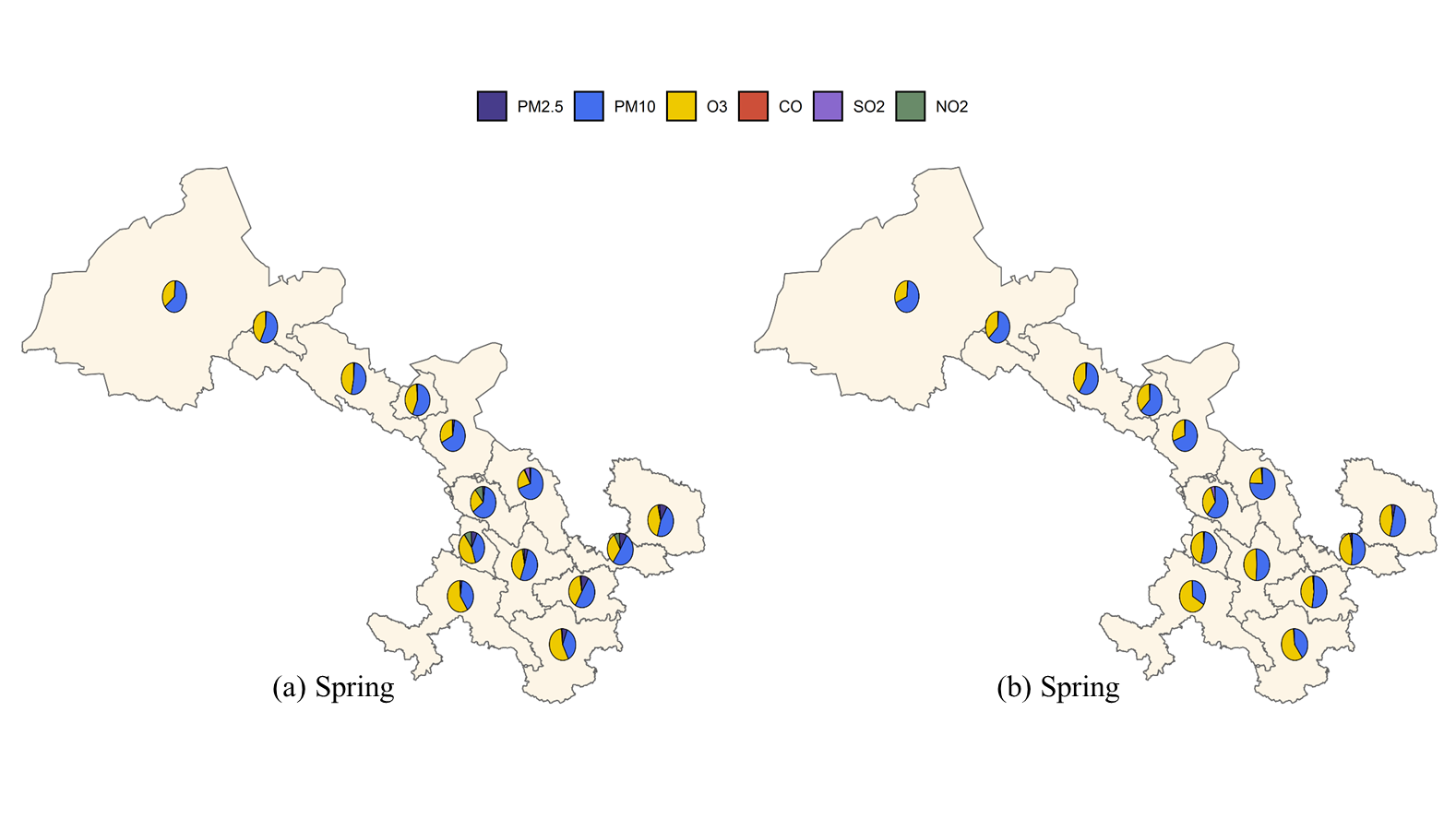


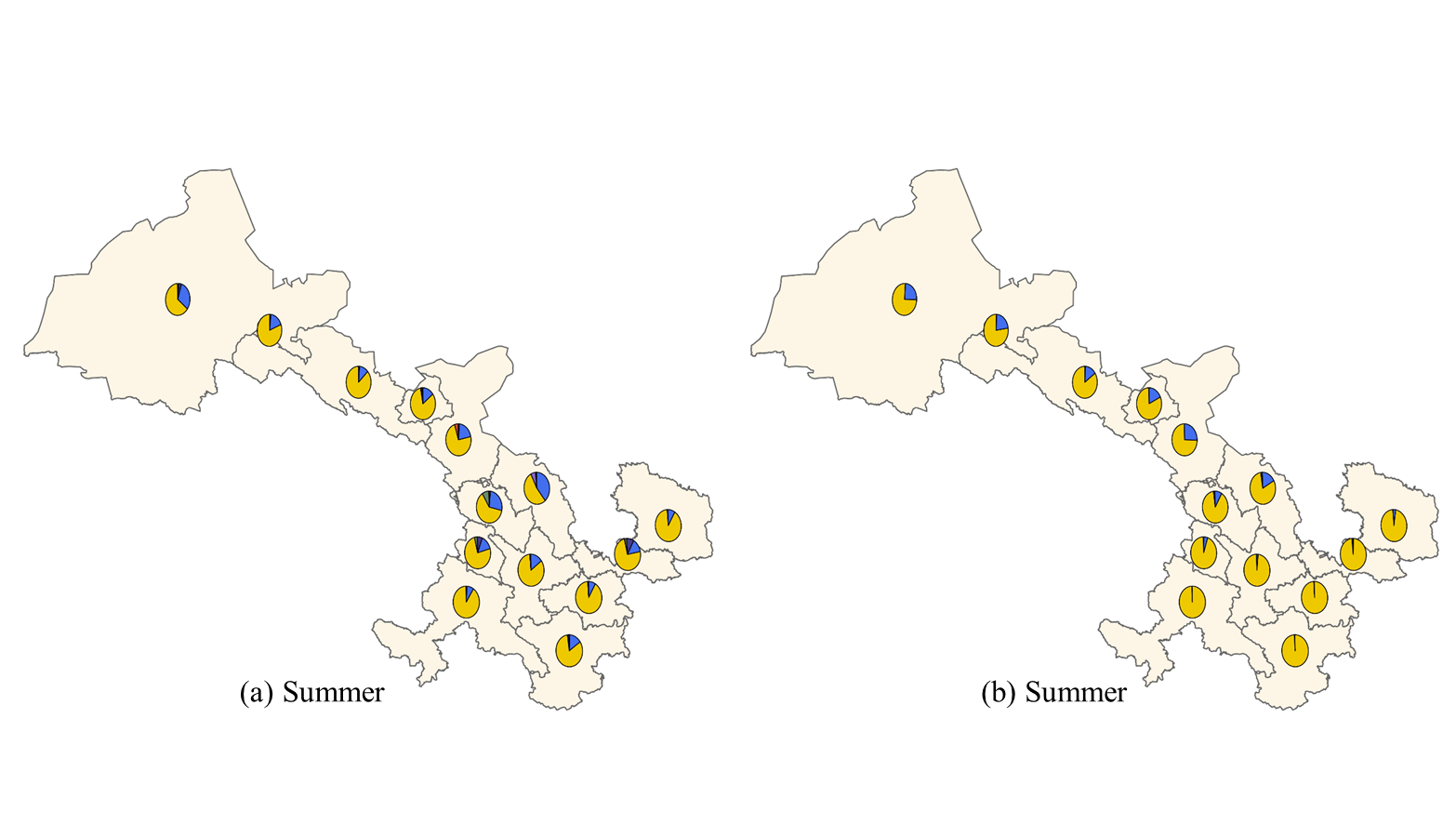


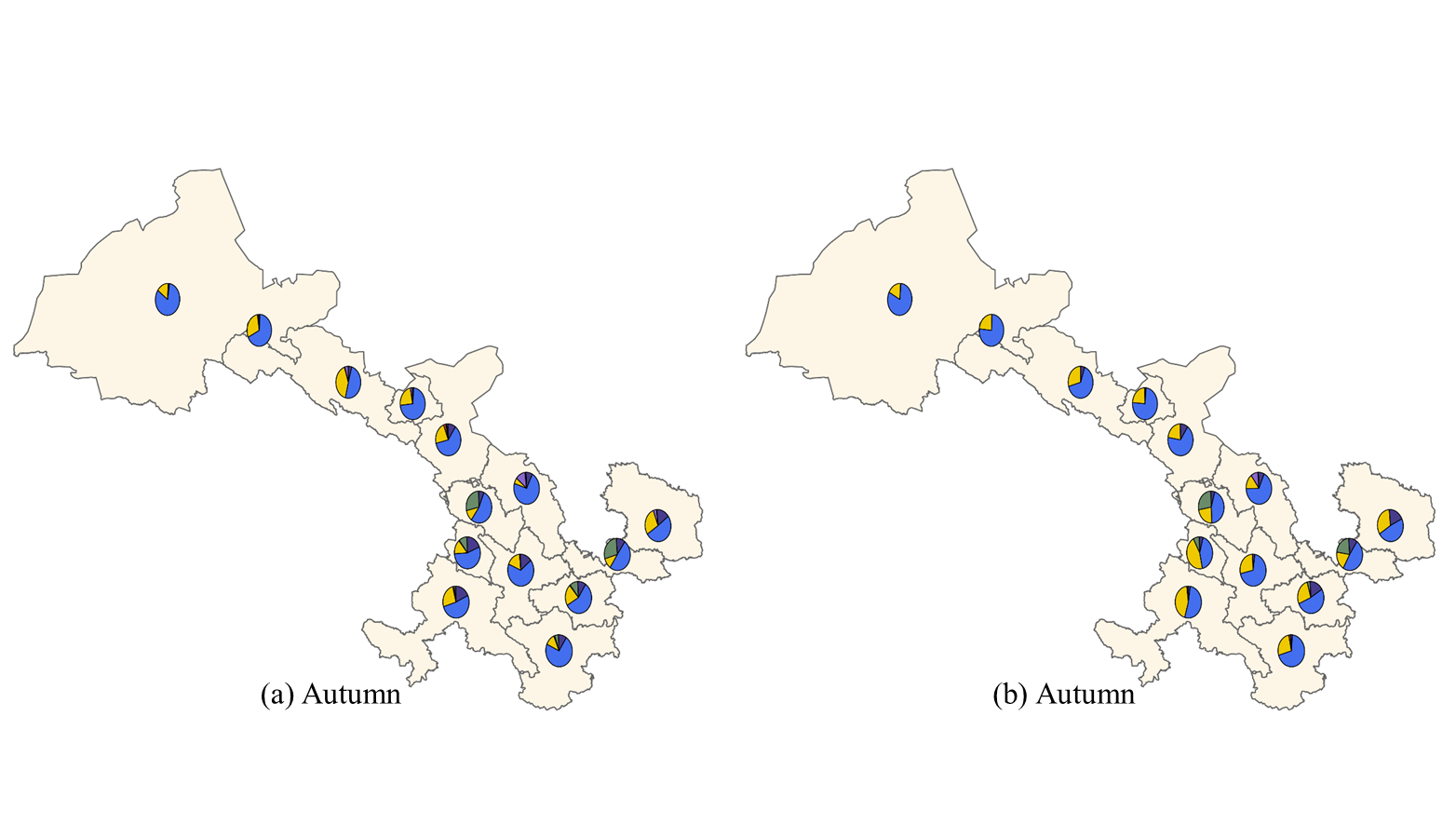


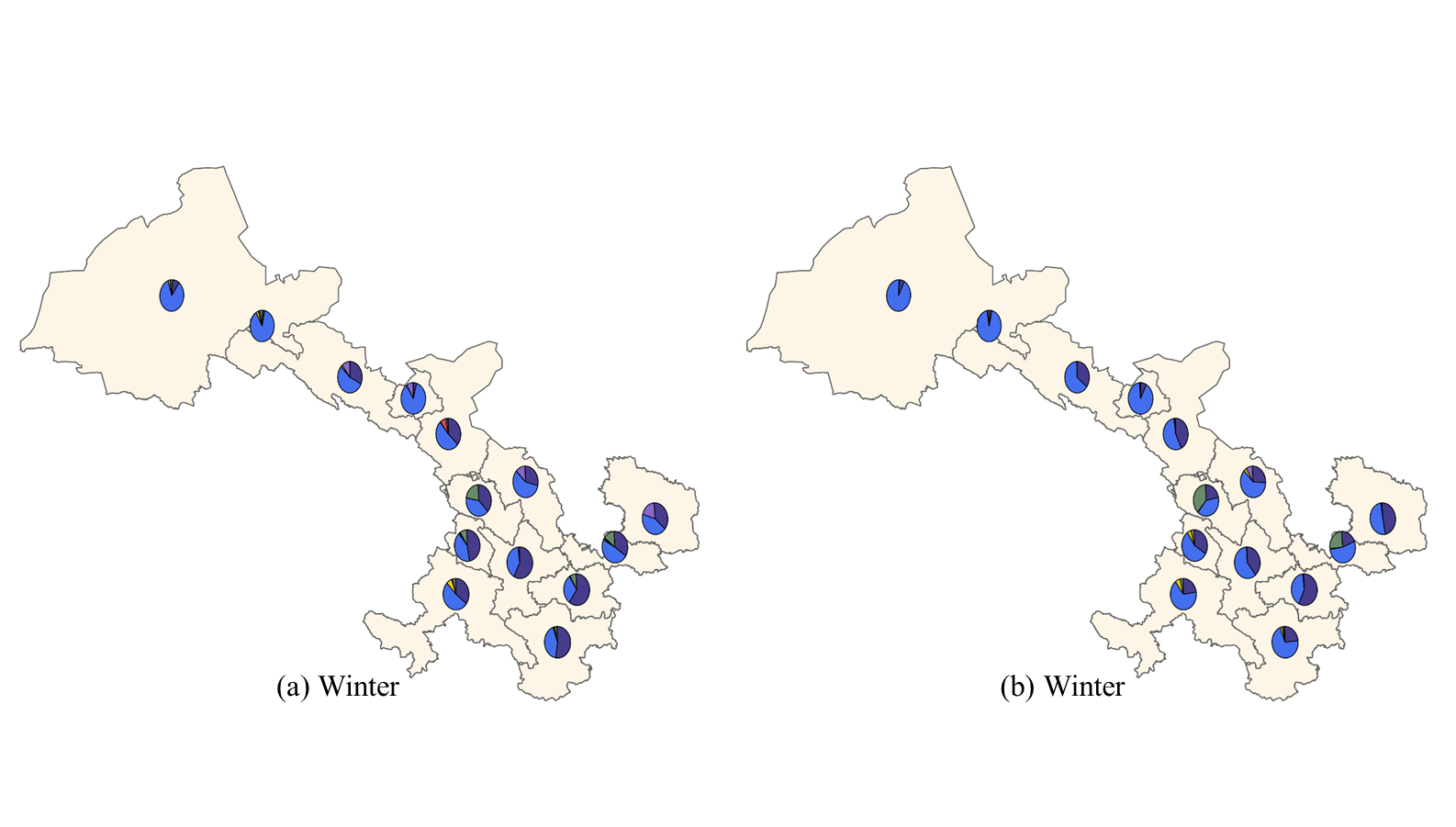


Fig S1. Seasonal distribution of major pollutant fractions in Gansu (a for P-I; b for P-II) (R version 4.0.1 https://www.r-project.org/)


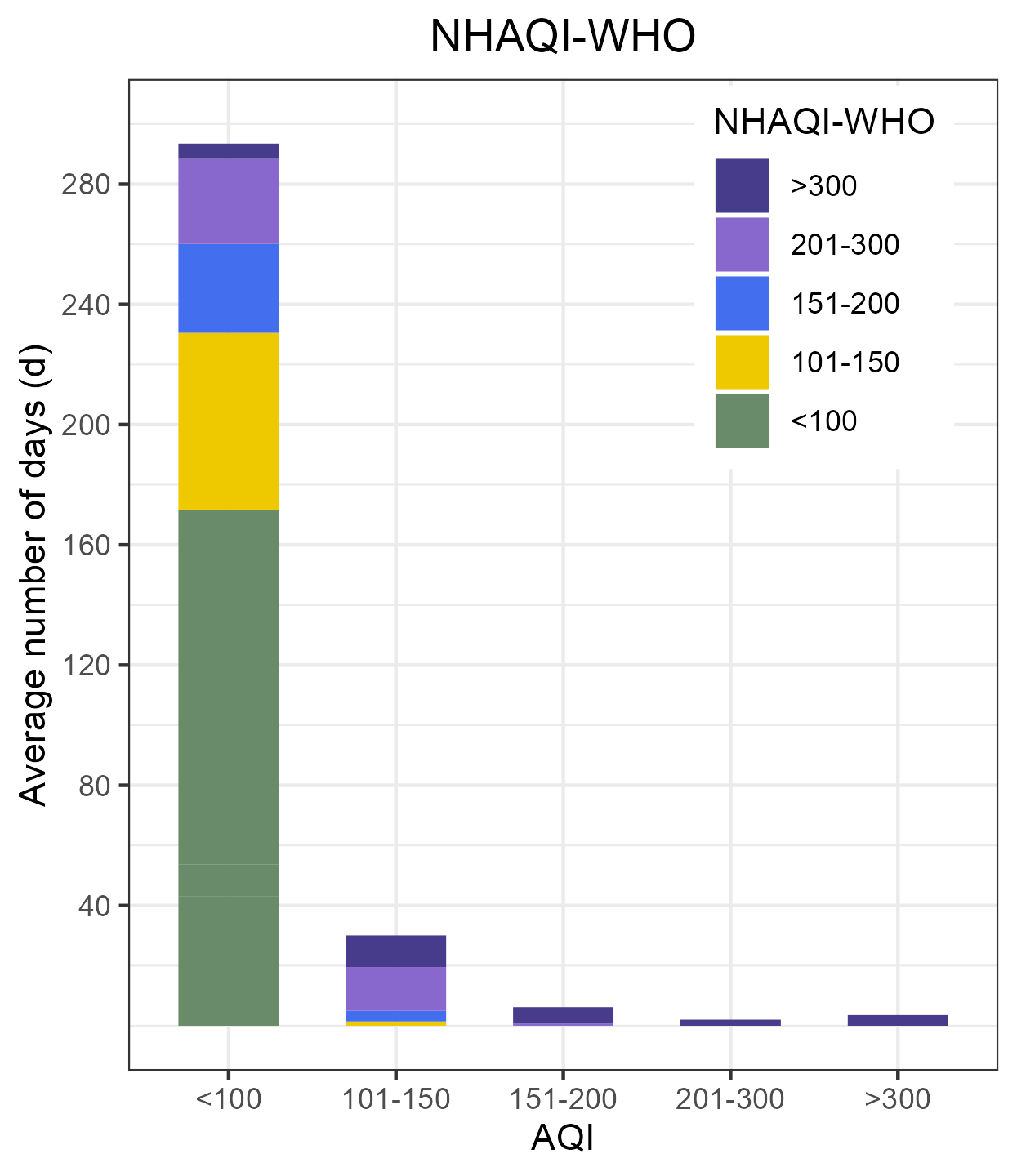


Fig. S2. Comparisons of NHAQI-WHO-based classifications of health risk type with the health type of AQI


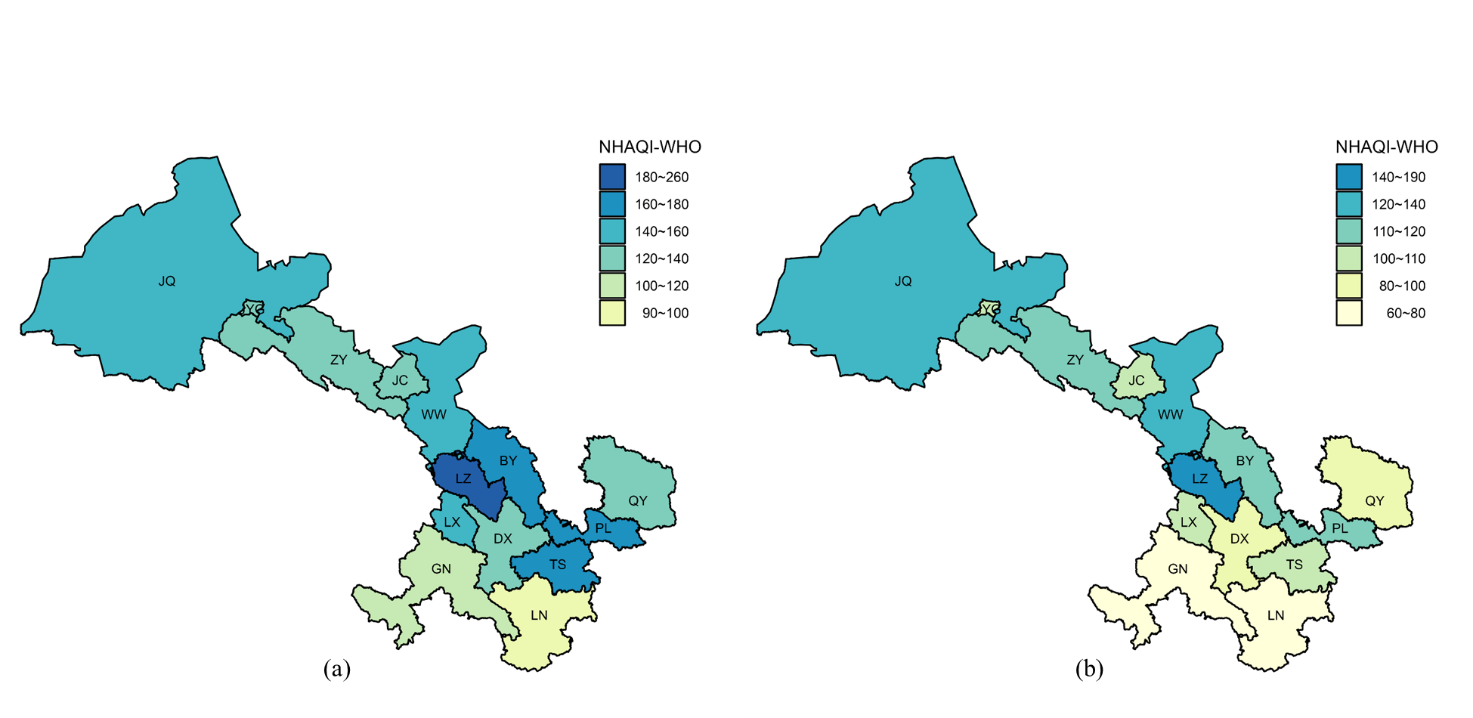


Fig. S3. Regional variations of mean NHAQI-WHO in Gansu (a for P-I; b for P-II) (R version 4.0.1 https://www.r-project.org/)


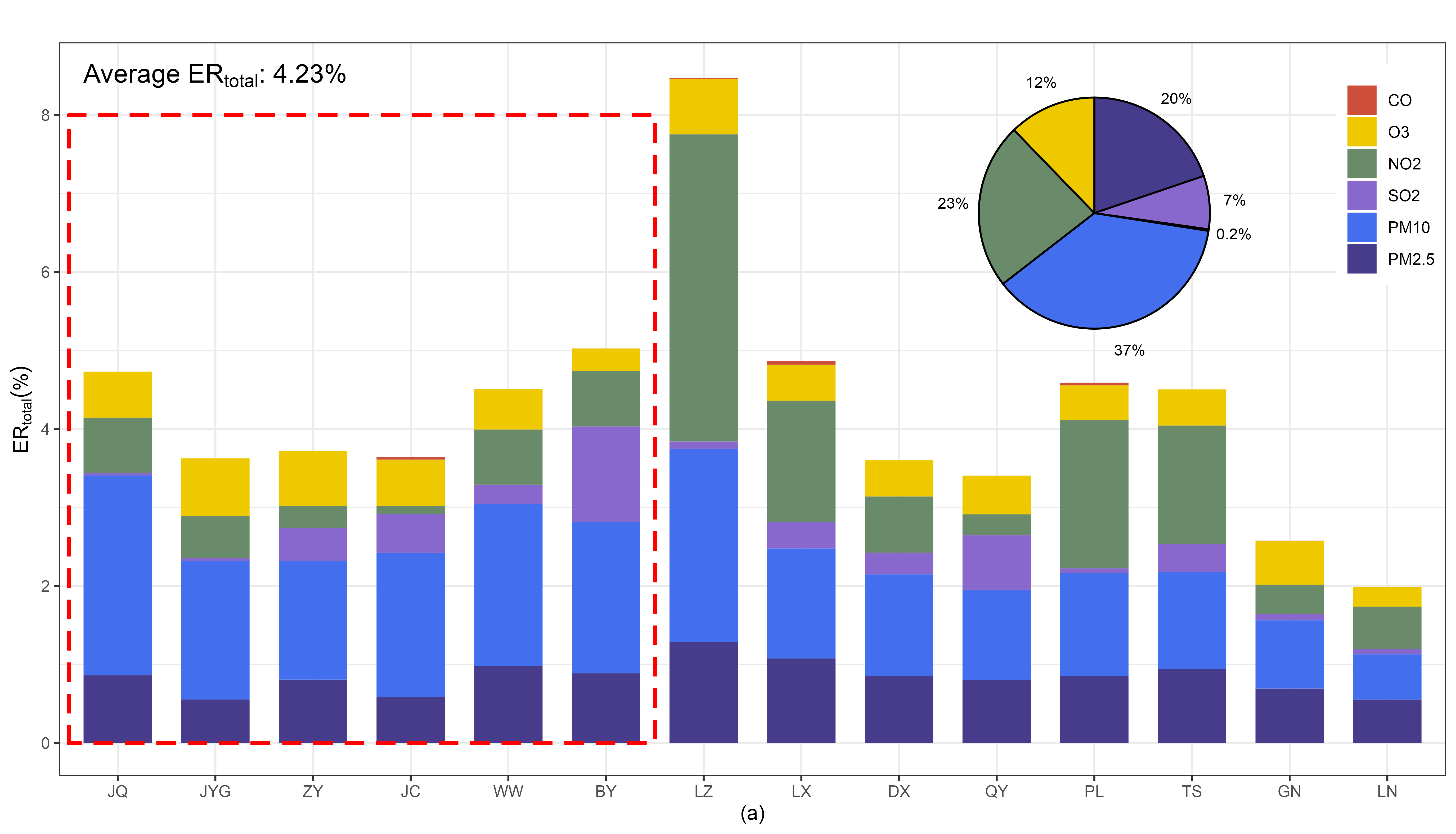


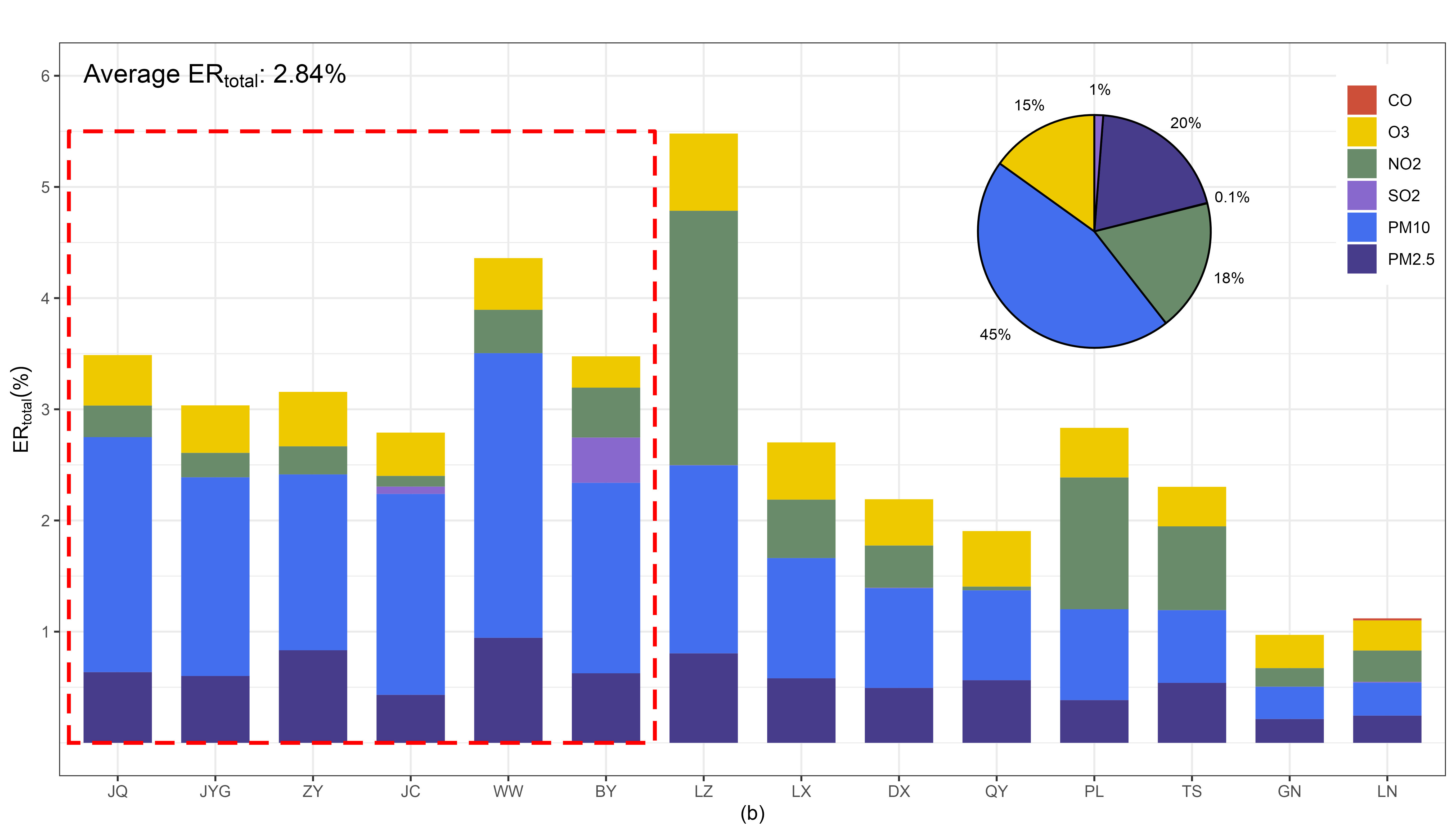


Fig. S4. Average ER_total_ in Gansu based on WHO standards (a for P-I; b for P-II)


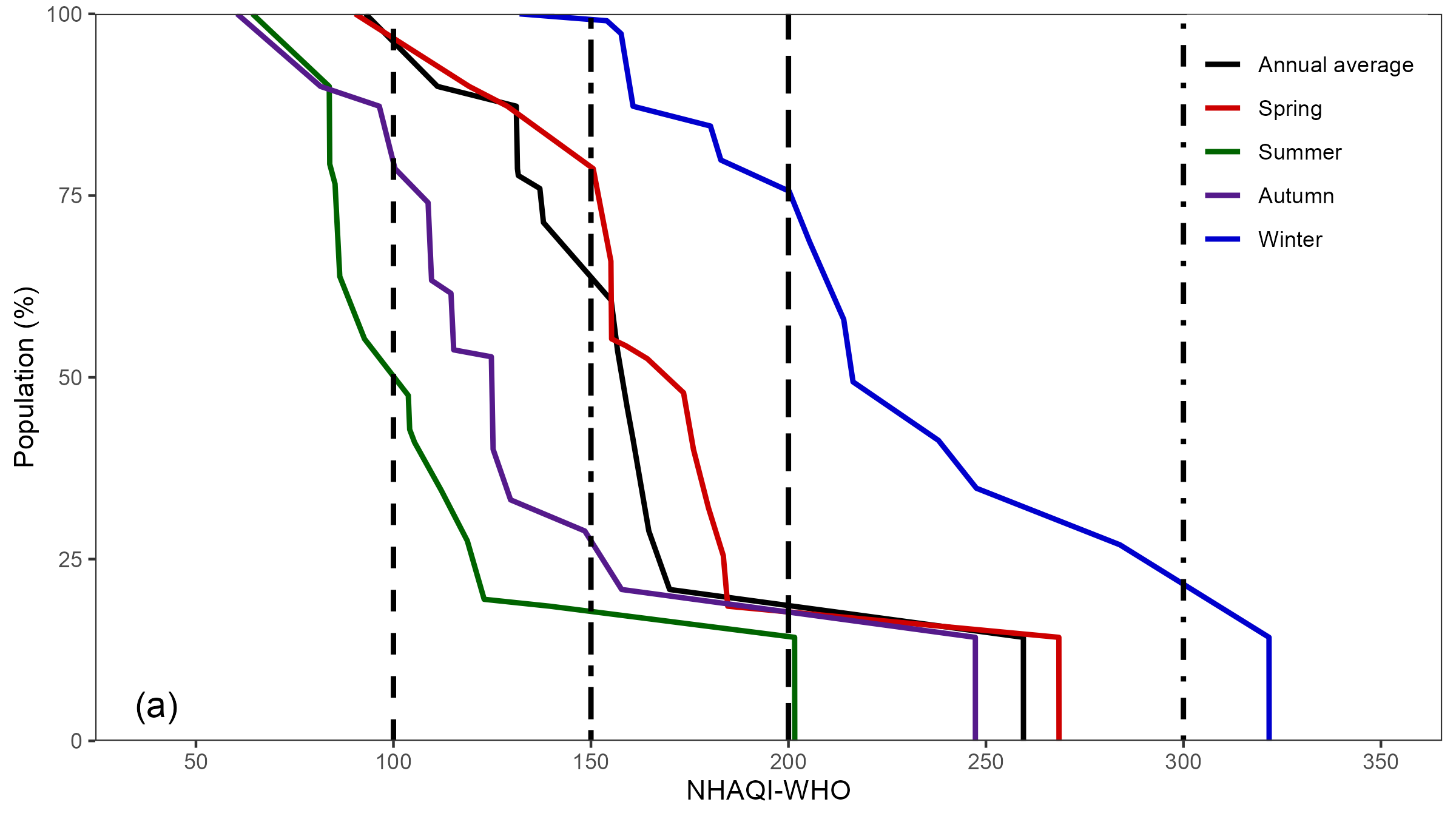

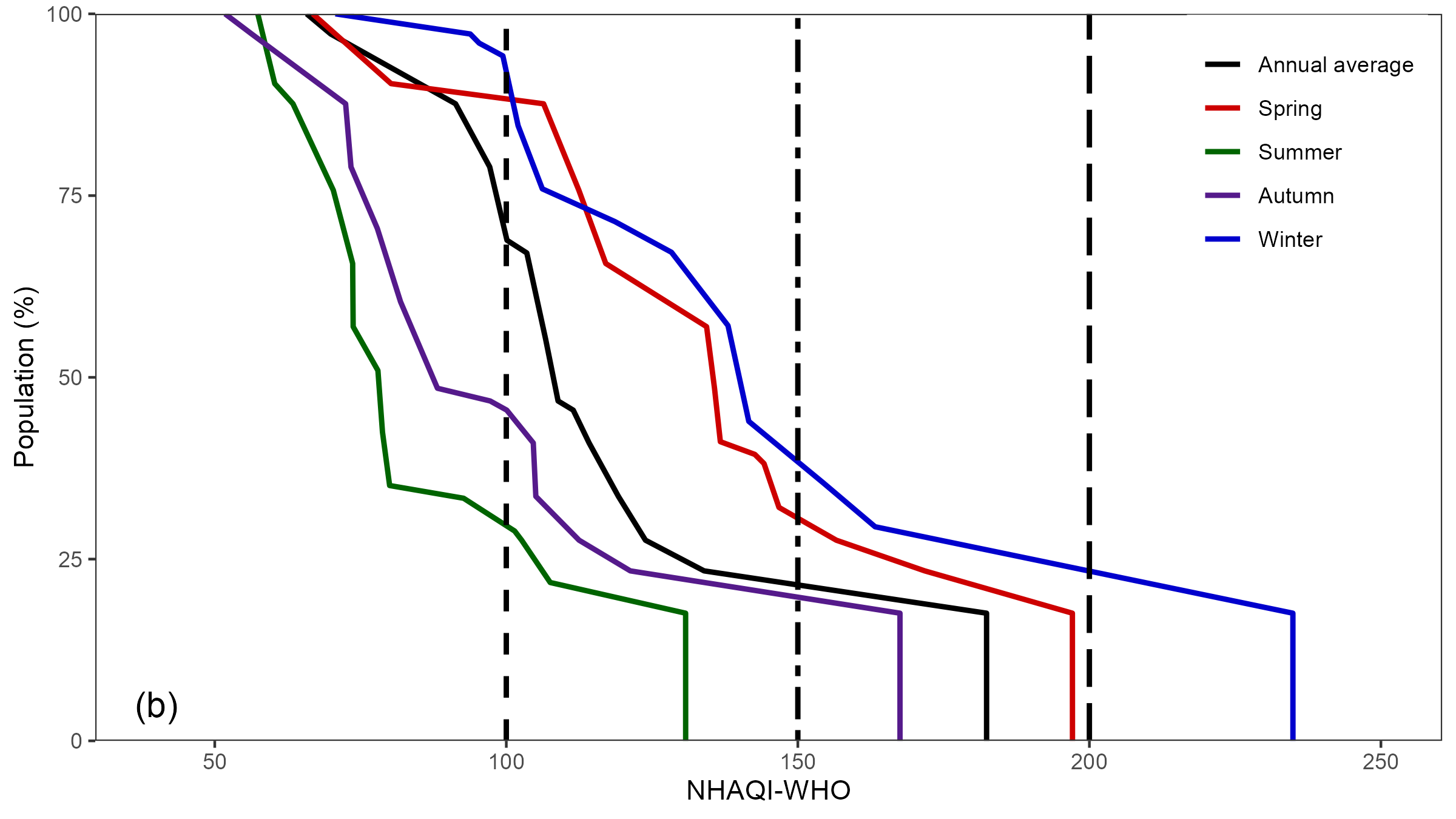


Fig. S5. Cumulative distribution of population-weighted estimations based on average NHAQI-WHO in Gansu (a for P-I; b for P-II)


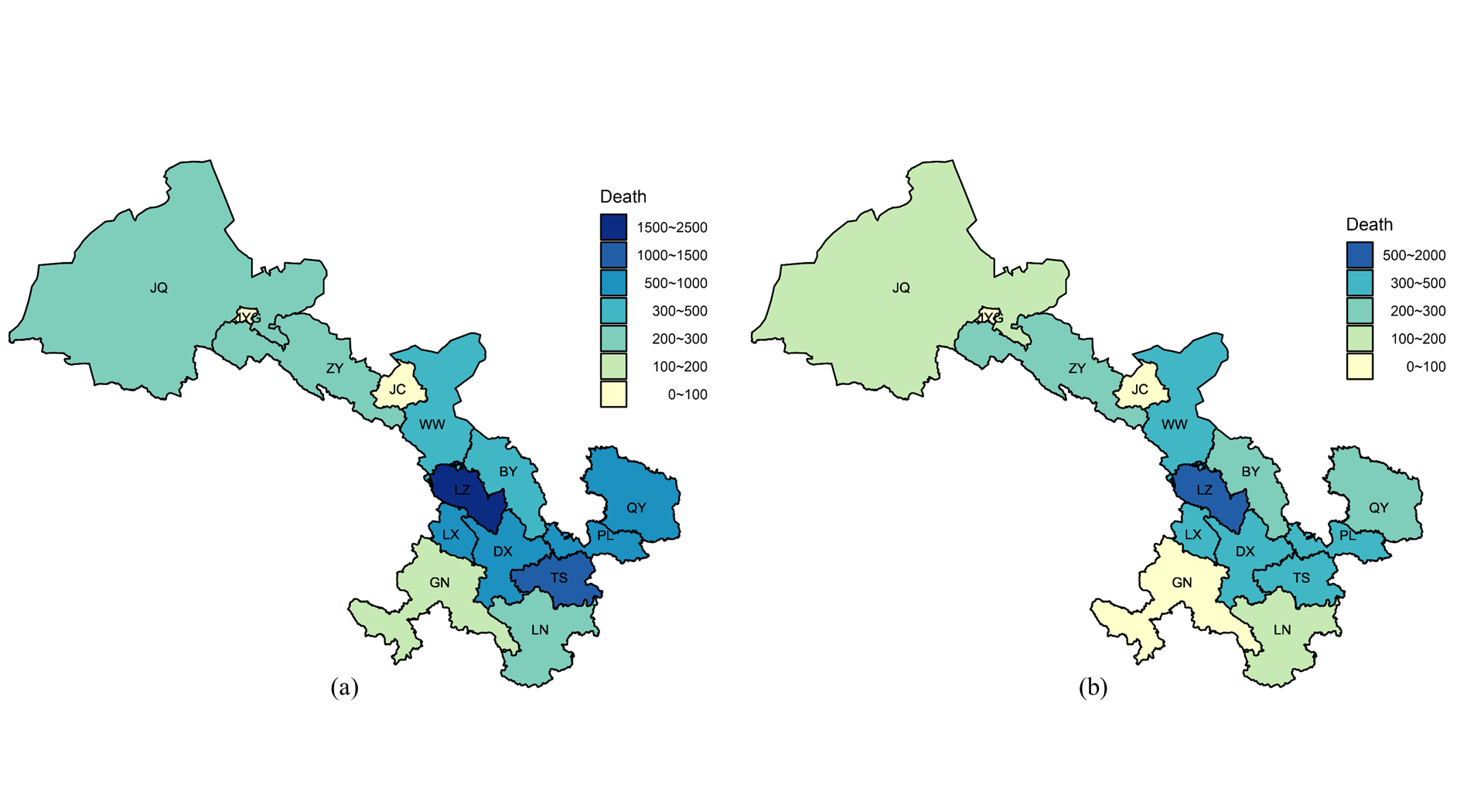


Fig. S6. Regional variations of the annual average deaths due to air pollution in Gansu based on WHO standards (a for P-I; b for P-II) (R version 4.0.1 https://www.r-project.org/)
